# Supplementary material for: Performance and scaling behavior of bioinformatic applications in virtualization environments to create awareness for the efficient use of compute resources
Source: PLoS Comput Biol. 2021 Jul 20;17(7):e1009244. doi: 10.1371/journal.pcbi.1009244 (PMC8323933; doi:10.1371/journal.pcbi.1009244)
Supplement: Attachment — Submitted filename: rebuttal_letter.rtf [file pcbi.1009244.s001.rtf]

Dear editor in chief, dear reviewers,
we thank you for the valuable remarks on our submitted paper with the title 
„Performance and scaling behavior of bioinformatic applications in virtualization environments to create awareness for the efficient use of compute resources“

We have gone carefully through the suggestions of the reviewers and would like to specify the changes made with respect to the individual remarks.

Tools:
We have added four more tools to the toolset (BBMap, BWA, MAFFT, SINA). These tools cover the sequence alignment category (BBMap, BWA) and the multiple sequence alignment (MAFFT, SINA) category. Further a re-run with 5 replicas for each tool has been conducted. Both remarks were made by reviewer 2.
For the categories Machine Learning and Molecular Dynamics we did not add new tools because with TensorFlow one of the most used and popular frameworks is already integrated, complemented with CIFAR-10 which is one of the most used test applications. Besides GROMACS, there are of course other tools but after years of experiences with molecular dynamics simulations it can be said, it is nearly impossible to run the same simulation with different tools with the same results. Therefore, we decided to use a tool that covers at least a large user community but also named other representatives. 
To make the tools selection more transparent, a new section (Tool selection) has been added, describing the reasons why a tool has been chosen, as noted by reviewer 1 in remark 2. 

Experimental setup:
Further, we explained the experimental setup in more detail (cf. section: System environment), especially providing more information on the dockerized version of BOOTABLE, Docker, OpenStack and the virtual environment (ratio of virtual to physical cores) as requested by reviewer 2 and 1 (note 2). 
An exemplary run of Docker on bare metal has been performed (cf. section: Performance measurements of virtual environments). A comment on the reason, why this scenario might not be of higher interest can be found in the Discussion and Analysis section (requested by reviewer 2).

Generalizability:
To improve the value of this study, an additional section with the title „Generalizability“ has been added to the Discussion and Analysis chapter, discussing under which conditions, findings can be generalized and applied to other tools, implementations or algorithms. To make the generalizability part more comprehensible for the readers, we have added Table 2 in the Tools section.
The table contains information about the used algorithms of a tool and or the used data structure. Thus we have followed the suggestions of reviewer 1 (notes 2, 3, 4) and reviewer 2 to make the general findings more comprehensible.

In general, the Tools section has remained the same, as Table 2 provides the additional information, suggested by reviewer 1. Further, we decided to explain the tools first and afterwards the datasets as this seems to be more reasonable from our point of view, regarding the reading flow, reviewer 1 (note 4, 5).

Datasets:
The Datasets section has been revised according to the remarks of reviewer 2. The categorization of the datasets into small, medium, large seemed to be unorthodox, so we changed it to the categories short, middle and long, referring to the expected runtime, a specific dataset will generate. We want to stick to a categorization in general, as this is relevant for the question, whether the size of a dataset has an impact on the scaling behaviour or not.
It is correct, that the different datasets are more or less in the same range of Megabytes, but small changes in the size of a dataset can have a large impact on the runtime, as most of the algorithms used by the tools are dependent on the input specifics. 
Further, it is correct that for some tools (Velvet and SPAdes) the same datasets are used. As we wanted to provide a smallest possible number of different datasets, we decided to use them for both categories (short, middle), as the generated wall-times are in a range between short and middle. We highlighted this in the corresponding part of the Datasets section. For the alignment with Bowtie2 we also use the same dataset for short and medium but against a different reference genome. We clarified this also in the corresponding section of the dataset.

Smaller corrections: 
We have made clear that machine learning belongs to the field of AI and is not part of the field of bioinformatics, although it is frequently used and finds applications in the bioinformatics community to answer different research questions (cf. section: Abstract), as remarked by reviewer 1 (note 4).
Regarding the remark of reviewer 2 on the „fluffiness“ of several parts, we tried to condense the content as much as possible, but specifically for the Tools section we had to take care that the algorithmic parts are still sufficiently represented, as they are used in context of the generalizability. Furthermore, we have taken care that Table 1 now appears closer to the point of its first mention

Figures: 
The figures showing the scaling plots, have been enhanced by a graphical illustration, showing an outlier interval (gray area). 
The red frame from Figure 1 has been removed.
The legends of Figures 3, 4, 5 (scaling plots) have been extended to provide more information, including the used datasets, as suggested from reviewer 1 (note 5) and 2. 
We also integrated the figures more tightly into the Results and Discussion chapter as reviewer 1 noted (note 5)
The remark that the figures' resolution is too low is difficult to resolve as the images have gone through the PACE pipeline, thankfully provided by the PLOS Journal. We assume that the .pdf provided to the reviewers does not necessarily contain the high resolution figures.

Tables: 
Table 4 has been removed as suggested by reviewer 2. 
We did not follow the suggestion to remove the comparison between the environments VM and Docker, as this would remove interesting results for the readers. It is not only of interest how a virtual environment performs against a bare metal setup. Many users are using cloud environments and applications in Docker containers on top, especially in the case of tools with large dependencies or complex installations. So we think that these information are of interest.
Further, it was not possible to transform Table 6 (now 7) as suggested from reviewer 2, as the length of the table would break the page limit, due to the four added tools.

Paper presentation: 
We have gone through the manuscript carefully to find and correct any typos, linguistic errors, redundant information (overlapping of sections „BOOTABLE“ and „System environment“), references and table locations.
We hope the language is more readable now and meets the remarks of reviewer 1 (note 5) and reviewer 2. We thank reviewer 2 for listing the linguistic errors and their corrections.
