# Supplementary material for: Performance and scaling behavior of bioinformatic applications in virtualization environments to create awareness for the efficient use of compute resources
Source: PLoS Comput Biol. 2021 Jul 20;17(7):e1009244. doi: 10.1371/journal.pcbi.1009244 (PMC8323933; doi:10.1371/journal.pcbi.1009244)
Supplement: Attachment — Submitted filename: rebuttal_letter_revision2.docx [file pcbi.1009244.s002.docx]

Dear editor in chief, dear reviewers,

we thank you for your additional remarks on the revised version of our manuscript with the title "Performance and scaling behavior of bioinformatic applications in virtualization environments to create awareness for the efficient use of compute resources".

We have gone carefully through the questions and remarks of the reviewers and would like to answer their questions and specify the changes made with respect to their remarks.

Results:

The results for the overhead numbers in Table 6 (page 18) differ from the values of the initially submitted manuscript. The differences can be explained by the newly added tools, in particular the SINA tool, which is responsible for the significant increase of the overhead values for the Docker variant. The deviation of the other overhead values results also from the newly added tools. Further, the increased number of replicas (from 3 to 5) reduced the impact of outliers to the lower end compared to the initial manuscript. Nonetheless, the overall picture remains the same although the absolute values have changed.

As indicated by reviewer 1, the classification of the tool IDBA in Table 7 (page 19), has changed from the categories "Yes" and "Partially" to "Partially" only. The change was made based on the achieved results for the revised version. In the initial version of the manuscript IDBA showed only a good scaling behavior for the long dataset, but was very close to the "Partially" category. The new data, including a higher number of replicas, show that the scaling behavior belongs more to the "Partially" category for the long dataset as the wall times increase with higher numbers of CPU cores at the end.

Tools description:

We have shortened the paragraphs describing the different tools in the Tools section to make the text more compact, as asked by reviewer 2. Still a minimum of stand-alone-information about each tool should remain to give readers from other research domain at least an initial idea.

Dataset description:

We added insights to the Dataset section (page 13) describing the impact on the wall times for the used datasets in general (requested by reviewer 2) and added the number of sequences, images or atoms to Table 3 (page 14) to make the correlations more transparent for the reader.

Regarding the general remark of reviewer 2 about the scope of the paper and the lack of new biological insights, we think it is a question about the interpretation of “and”. One can see it in a strict and logical way, concluding that each manuscript must fulfill all five categories mentioned in the PLOS Comp. Bio. Scope. We hope that you share our more liberal view interpreting the “and” as list separator, consequently interpreting the five mentioned aspects as reference point within the high dimensional scientific research space. We see our paper as contribution to the methods realm hopefully helping to facilitate future scientific insight.

Sincerely yours,

Max Hanussek and Jens Krüger
